# Unrelenting Vision Loss: The Virulence of Klebsiella pneumoniae

**DOI:** 10.7759/cureus.44786

**Published:** 2023-09-06

**Authors:** Minali Prasad, Tova Kosowsky, Xuejing Chen, Samaneh Davoudi Moghaddam, Steven Ness, Crandall Peeler, Nicole H Siegel, Manju L Subramanian

**Affiliations:** 1 Ophthalmology, Boston University Chobanian & Avedisian School of Medicine, Boston, USA; 2 Ophthalmology, Boston Medical Center, Boston, USA

**Keywords:** intravitreal antibiotic, subretinal abscess, endophthalmitis, klebsiella endophthalmitis, klebsiella pneumoniae

## Abstract

A 37-year-old Hispanic male with a recent history of COVID-19 infection and type 2 diabetes mellitus was admitted to the hospital with shortness of breath, chest pain, and hyperglycemia. Eye exam and imaging findings indicated endogenous endophthalmitis confirmed by blood cultures that speciated to *Klebsiella pneuomoniae*. The patient’s eye condition progressed, ultimately resulting in no light perception less than a month after the initial evaluation. Due to the rapidly progressive nature of Klebsiella endogenous endophthalmitis, we recommend that primary teams consult ophthalmology for close monitoring of patients with a high index of suspicion.

## Introduction

*Klebsiella pneumoniae*, which is a gram-negative, anaerobic, rod-shaped bacterium, is a rare cause of endogenous endophthalmitis [1,2], classically preceded by the formation of liver abscesses. While one of the characteristics of hypervirulent *Klebsiella pneumoniae* is its ability to cause metastatic infectious disease in hosts that are otherwise relatively healthy, the major underlying systemic risk factor in these cases has been identified to be diabetes mellitus [3,4]. In the present report, we describe a rapidly progressive case of *Klebsiella* endophthalmitis with subretinal abscesses that resulted in devastating vision loss.

## Case presentation

A 37-year-old Hispanic male with a medical history of type 2 diabetes mellitus (hemoglobin A1c 10.9) and a COVID-19 infection that occurred three weeks before the presentation was hospitalized with symptoms of hyperglycemia, shortness of breath, chest pain, and a new oxygen requirement. His symptoms were initially attributed to prolonged COVID-19 infection with superimposed community-acquired pneumonia, and he was treated for his acute hypoxemic respiratory failure with steroids and ceftriaxone/azithromycin with daily monitoring of blood cultures due to vital instability. The patient reported a change in vision in the right eye three days into admission and requested an ophthalmology consultation.

Upon initial evaluation, the patient reported blurry vision with a "smoky black" veil in his right eye, a significant headache, and binocular diplopia. He denied experiencing floaters, flashes, or ocular pain in the right eye as well as any symptoms in the other eye. His uncorrected near visual acuity was 20/40 and 20/25 in the right and left eyes, respectively. The examination of his pupils, which were small and minimally reactive to light and accommodation, was confounded by morphine administration. The intraocular pressure (IOP) was 8 mmHg and 7 mmHg in the right and left eyes, respectively. The patient also had mild intermittent exotropia with full ocular motility. A bedside examination revealed a posterior subcapsular cataract in the right eye and otherwise normal anterior segment bilaterally. Moreover, a dilated fundus examination showed macular edema with exudates and scattered intraretinal hemorrhages in the right eye and a normal appearance in the left eye. No other diagnostic testing was obtained given the patient's inpatient status requiring oxygen supplementation, and the fundus findings were attributed to asymmetric, mild, non-proliferative diabetic retinopathy.

During the evening of the ophthalmologic evaluation, the patient acutely decompensated and required intubation as well as a transfer to the ICU. The blood cultures drawn earlier that morning grew gram-negative rods (within 24 hours of incubation), speciating to *Klebsiella* the following day. The patient was diagnosed with disseminated *Klebsiella* bacteremia complicated by pneumonia, with imaging and lumbar puncture supporting secondary diagnoses of liver abscesses and *Klebsiella* meningitis, all of which were consistent with a hypervirulent strain of *Klebsiella*. Consequently, he was started on the systemic central nervous system (CNS) dosing of intravenous ceftriaxone and underwent interventional-radiology-guided drainage of his liver abscesses.

**Figure 1:**
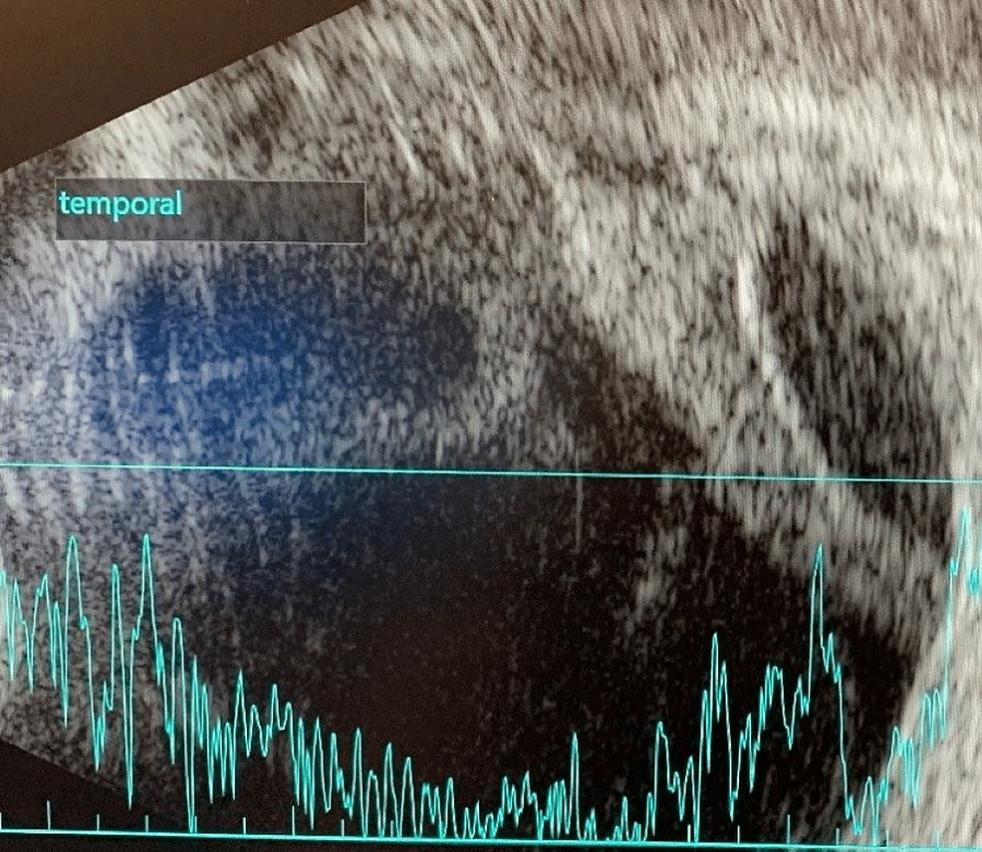
Initial B-scan (hospital day four) with vitreous debris and temporal subretinal abscess formation

**Figure 2:**
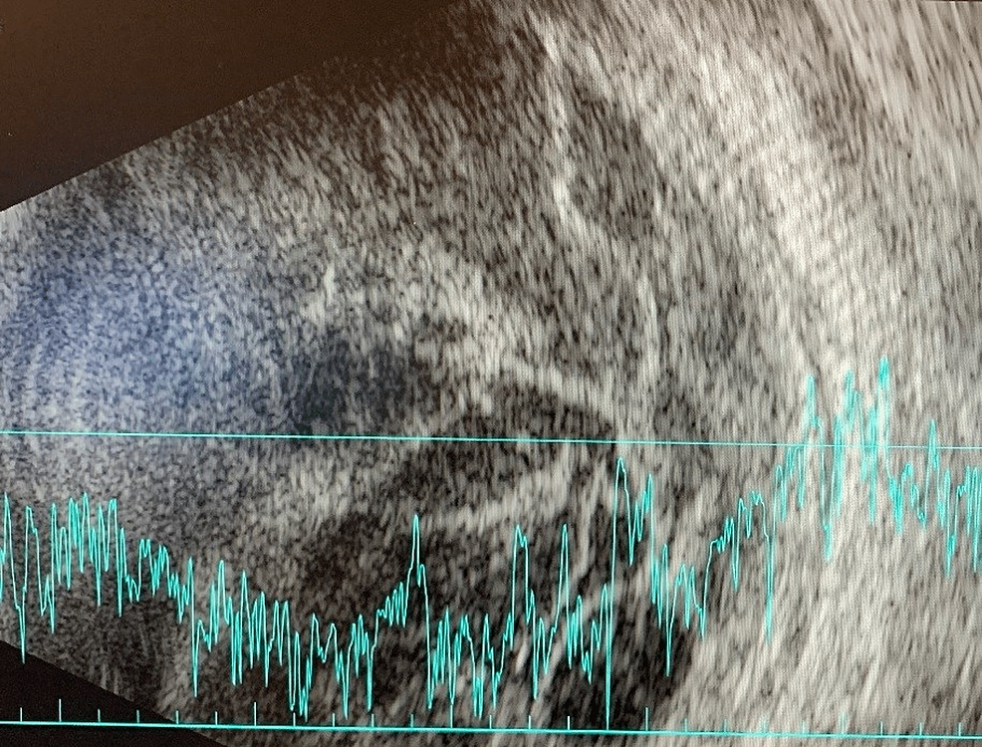
B-scan (hospital day 11) with worsening subretinal abscess growth, loculations, and vitritis

Three days following his initial eye examination, the patient developed conjunctival injection and chemosis while maintaining a clear cornea and quiet anterior chamber. A limited fundus examination showed a hazy, white reflex with inferonasal white-centered retinal hemorrhages. B-scan ultrasound showed loculated vitreous debris with a temporal subretinal abscess collection (Figure 1). There was immediate concern for endogenous endophthalmitis. A vitreous tap was performed at this time, four days following the initiation of systemic ceftriaxone, and the patient received intravitreal injections of vancomycin and ceftazidime. Atropine and moxifloxacin eye drops were started, and he was continued on systemic high CNS dosing of IV ceftriaxone. Though his vitreous sample had a negative gram stain without subsequent fungal or bacterial growth, a repeat B-scan from two days later demonstrated stable vitritis with an increasingly loculated subretinal abscess (Figure 2). Further, the patient continued to receive intravitreal injections every two days while intubated, which were later modified by the discontinuation of vancomycin and the addition of amikacin for the double coverage against *Klebsiella* 13 days following his initial injection.

**Figure 3:**
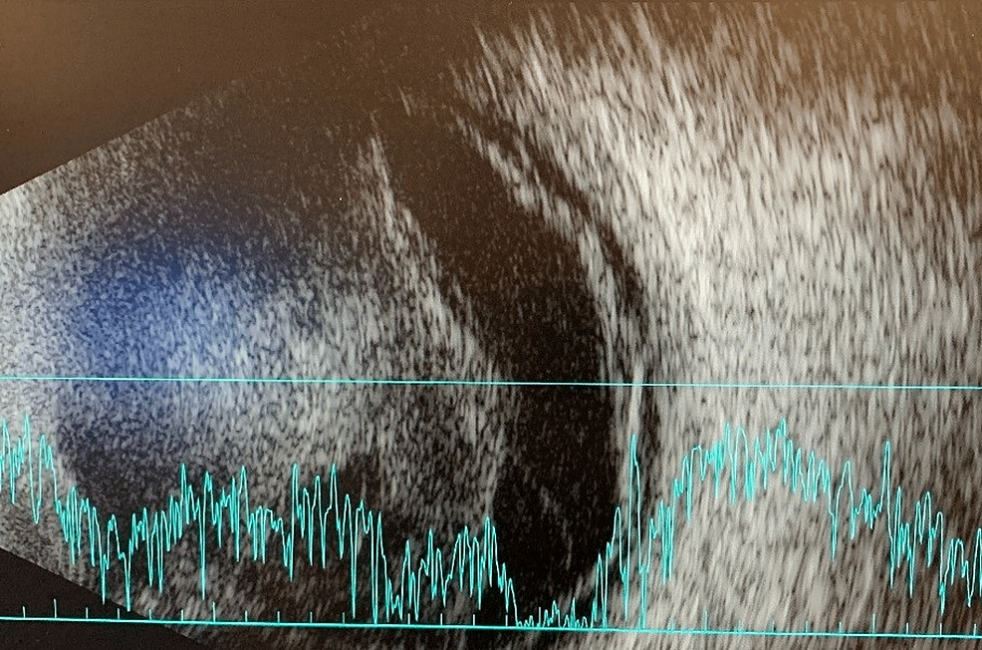
B-scan (hospital day 23) with diffuse, near-total shallow retinal detachment

**Figure 4:**
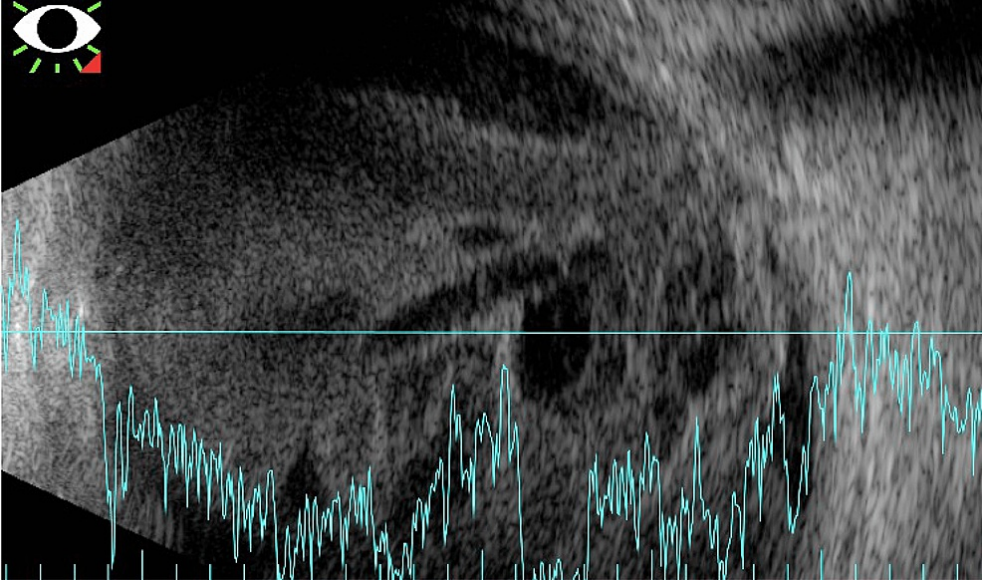
B-scan (hospital day 23) demonstrating the area of retinal attachment at the optic nerve; diffuse vitreous debris with loculated collections are present

A week following his acute decompensation, the patient was successfully extubated, at which time he reported decreased vision with increased pain, flashes, and floaters in his right eye. A slit lamp examination on hospital day 10 showed visual acuity of light perception (LP) with a new layered hypopyon and diffuse fibrin, and the patient was started on prednisolone eye drops. On hospital day 24, the patient's vision worsened to no light perception (NLP), and while there was no fundoscopic view on clinical examination, a B-scan showed a shallow, near-total retinal detachment (Figures 3, 4). The goals of care were discussed, and a decision was made to focus on comfort measures for the right eye.

## Discussion

While the majority of patients with *Klebsiella* endophthalmitis evaluated by ophthalmologists are found to have initial visual acuities of counting fingers or worse [5-8], the present case was unique in that ophthalmologists were consulted before the disseminated infection and the rapid deterioration of vision. Our patient's acute decompensation was most likely multifactorial and secondary to his history of diabetes and COVID-19, as well as the virulent nature of *Klebsiella*. Infections from emerging hypervirulent strains of *Klebsiella pneumoniae* appear to follow a defined clinical course with liver abscesses as the first sign of infection, followed by metastatic spread manifesting as endophthalmitis, meningitis, necrotizing fasciitis, and abscesses of the central nervous system, psoas, and prostate [9]. At present, the definition of hypervirulent *Klebsiella* is not fully elucidated, but multiple virulence factors and phenotypes have been identified as markers of these hypervirulent strains, with the classic clinical picture being highly suggestive [10,11]. While rare, this hypervirulent strain of *Klebsiella* *pneumoniae*, especially in the setting of diabetes, is the most common cause of endogenous endophthalmitis in Asian countries and is spreading globally, with cases being reported in the United States, Australia, and across Europe [8,12]. While we did not have positive eye fluid cultures to confirm dissemination of *Klebsiella* into the patient's eye, the rapid incubation period, positive blood culture, the patient's clinical course, and the findings of liver abscesses, followed by endophthalmitis and meningitis, are consistent with previously described manifestations of hypervirulent *Klebsiella* endophthalmitis.

Among the patients for whom visual acuity improved, the common treatment was systemic antibiotics such as intravenous gentamicin, clindamycin, and ceftriaxone, in combination with intravitreal antibiotics (including various combinations of gentamicin, cefazolin, and vancomycin) [5-8]. While there has been neither an established protocol for the timing of surgery with pars plana vitrectomy (PPV) nor strict surgical indications in patients with *Klebsiella* endophthalmitis, clinical trends suggest that the earlier intervention with PPV may carry a more favorable visual prognosis. A retrospective review of 10 eyes suggested that earlier surgical intervention for *Klebsiella* endophthalmitis is associated with improved outcomes [13]. Surgical intervention has been reported to be beneficial, particularly among patients without clinical improvement with intravitreal antibiotics [13-15]. Indeed, the majority of bacterial endophthalmitis cases requiring enucleation in a prospective case series of 64 patients grew culture-positive *Klebsiella*, suggesting that early aggressive management, including early vitrectomy, may be justified [14]. Other effective anti-inflammatory treatments include systemic, topical, or intravitreal steroids, including dexamethasone [16]. The visual acuity of a 51-year-old male patient with *Klebsiella* endophthalmitis reportedly improved after treatment with PPV, systemic and intravitreal antibiotics, as well as systemic, topical, and intravitreal corticosteroids [15]. Supportive treatment may involve cycloplegic medications (for ciliary spasms and prevention of synechiae), IOP-lowering medications or vitreous taps, and topical hypertonic saline (for corneal edema) [16]. 

Subretinal abscesses in *Klebsiella* endophthalmitis may be surgically managed with PPV to improve visual outcomes. The drainage of a subretinal abscess during vitrectomy was found to restore vision in a patient with endogenous *Klebsiella* endophthalmitis [17]. There is a reported case of similar success when performing a vitrectomy in combination with intravitreal antibiotics for subretinal abscess drainage [18]. In the present case, while early vitrectomy and abscess drainage was considered and discussed for the patient, his unstable medical status made him an inappropriate surgical candidate. Unfortunately, by the time he was medically cleared for surgery, his vision worsened to NLP. B-scan ultrasonography demonstrated a near-total retinal detachment, likely secondary to a combination of vitreous traction and subretinal abscess growth, which ultimately led to retinal tears and subsequent detachment. A decision was made to focus on medical treatment with the ultimate goal of comfort measures for the right eye.

In contrast to previously published case reports, the patient in the present case had a bedside visual acuity of 20/40 upon initial consultation before the culture data, which, within six days of his first eye examination, worsened to LP. This rapid deterioration of vision was likely a function of the bacteria's quick generation time of 2.31 hours [19], which represents the amount of time for a population of bacteria to double in number [20]. The main prognostic factor in *Klebsiella* endophthalmitis is the presence of hypopyon, with other prognostic factors including rapid onset of ophthalmic symptoms, unilaterality, and panophthalmitis [3].

## Conclusions

Due to the rapidly progressive nature of *Klebsiella *endogenous endophthalmitis and the virulence of *Klebsiella *bacteremia, we recommend that primary and ophthalmology teams should monitor patients closely once blood cultures have confirmed the causative organism until source control has been achieved and bacteremia is cleared. These patients remain at extremely high risk of *Klebsiella* endogenous endophthalmitis, which is a devastating and blinding complication of this virulent organism.
